# Role of antioxidants in fertility preservation of sperm — A narrative review

**DOI:** 10.5713/ab.22.0325

**Published:** 2022-11-14

**Authors:** Ahmad Yar Qamar, Muhammad Ilyas Naveed, Sanan Raza, Xun Fang, Pantu Kumar Roy, Seonggyu Bang, Bereket Molla Tanga, Islam M. Saadeldin, Sanghoon Lee, Jongki Cho

**Affiliations:** 1 College of Veterinary and Animal Sciences, Jhang 35200, Sub-campus of University of Veterinary and Animal Sciences, Lahore 54000, Pakistan; 2 Laboratory of Theriogenology, College of Veterinary Medicine, Chungnam National University, Daejeon 34134, Korea; 3 Daejeon Wildlife Rescue Center, Chungnam National University, Daejeon 34134, Korea

**Keywords:** Antioxidants, Infertility, Oxidative Stress, Reactive Oxygen Species, Sperm

## Abstract

Male fertility is affected by multiple endogenous stressors, including reactive oxygen species (ROS), which greatly deteriorate the fertility. However, physiological levels of ROS are required by sperm for the proper accomplishment of different cellular functions including proliferation, maturation, capacitation, acrosomal reaction, and fertilization. Excessive ROS production creates an imbalance between ROS production and neutralization resulting in oxidative stress (OS). OS causes male infertility by impairing sperm functions including reduced motility, deoxyribonucleic acid damage, morphological defects, and enhanced apoptosis. Several *in-vivo* and *in-vitro* studies have reported improvement in quality-related parameters of sperm following the use of different natural and synthetic antioxidants. In this review, we focus on the causes of OS, ROS production sources, mechanisms responsible for sperm damage, and the role of antioxidants in preserving sperm fertility.

## INTRODUCTION

Male fertility can be negatively impacted by multiple exogenous and endogenous stressors including reactive oxygen species (ROS). ROS are produced during oxygen metabolism either owning to the electron transport chain system or different conditions associated with enhanced energy demands. The highly reactive nature of ROS enables them to react with and modify any molecule through oxidation resulting in structural and functional alterations [1]. The most common type of produced ROS includes superoxide anion radicals (O_2_^–•^), hydrogen peroxide (H_2_O_2_), and hydroxyl radicals (OH^•^).

Recent reports have shown that ROS plays an important role in both reproductive physiology and pathology. This dual nature of ROS depends on the source, concentration, production site, and exposure time [2]. The physiological level of ROS is considered important for the proper accomplishment of different functions associated with gamete fertility including proliferation, maturation, the release of oocytes [3], capacitation, hyperactivation, acrosomal reaction, and fertilization [4]. However, ROS overproduction can trigger pathological responses damaging cells and tissues.

Living organisms are equipped with natural defense systems (antioxidants) to scavenge and neutralize the effects of ROS. Reports have verified the presence of a wide range of antioxidants in the seminal plasma that can protect sperm against the detrimental effects of ROS [5]. The enzymatic antioxidants present in the seminal plasma include superoxide dismutase (SOD), catalase (CAT), and glutathione peroxidase (GPx), whereas, non-enzymatic antioxidants include vitamins A and C, carnitine, glutathione (GSH), and pyruvate [5].

Under physiological conditions, an equilibrium is maintained in the male reproductive tract between ROS production and neutralization. However, excessive ROS production can overcome the antioxidant defense systems resulting in oxidative stress (OS). OS has been reported as a major cause of sperm damage affecting fertility [2]. The plasma membrane of the mammalian sperm is rich in polyunsaturated fatty acids (PUFAs) that increase vulnerability to oxidative damage [1]. Moreover, sperm with limited cell repair machinery lacks significant antioxidant protection owning to the limited volume and restricted cytoplasm distribution in the sperm cells [5].

Recent findings have shown that OS is associated with an increased percentage of damaged sperm due to the oxidation of deoxyribonucleic acid (DNA), lipids, proteins, and nucleotides [6]. Ultimately, the structural integrity of the plasma membrane is lost along with reduced sperm motility, an increase in morphological abnormalities, and cellular apoptosis [7]. Therefore, OS leads to infertility through impaired sperm function. The seminal plasma of fertile individuals shows greater antioxidant capacity than that of infertile ones [8]. One approach to control OS-induced damage is through the use of antioxidants that act by scavenging and neutralizing ROS. Over the years, researchers have used different antioxidants (natural and synthetic) either alone or combined and at different dosages for varying durations. Several *in-vivo* and *in-vitro* studies have reported beneficial outcomes achieved following antioxidant use including enhanced sperm concentration and motility, reduced morphological abnormalities and DNA fragmentation, better antioxidant capacity of seminal plasma, and improved outcomes of assisted reproductive biotechnologies [9]. However, in some cases, *in-vitro* improved sperm quality failed to improve fertility in clinical trials. In this review, we focus on the sources of ROS in semen, physiological and pathological effects of ROS on sperm, and the role of endogenous and exogenous antioxidants in preserving sperm fertility.

## SOURCES OF ROS IN SEMEN

### Intrinsic sources

#### Sperm

Sperm are the primary source of ROS produced in the semen. However, the amount of ROS produced depends on the maturation stage of sperm. In mature sperm, ROS production occurs either in the plasma membrane by nicotinamide adenine dinucleotide phosphate (NADPH) oxidase or in the presence of a nicotinamide adenine dinucleotide (NADH)-dependent oxidoreductase in the inner mitochondrial membrane, which ensures ROS production through electron leakage from the electron transport chain [10].

#### Leukocytes

The leukocytes found in the semen are the second major source of ROS that acts as an integral part of the cellular defense system against infection, varicocele, spinal cord injury, and prolonged sexual absentia, and inflammation [10]. Leukocytic infiltration is enhanced during infection to counter infectious agents. Increased production of cytokines associated with inflammatory processes, such as interleukin-8, along with decreased production of SODs results in a respiratory burst and excessive ROS production leading to OS [11].

### Extrinsic sources

Excessive ROS production in the semen may be associated with certain extrinsic factors including the use of alcoholic drinks, air pollution, smoking, obesity, heat stress, toxicants or radiation exposure, aging, environmental factors, and nutritional deficiencies [12]. The ROS concentration in semen also depends on *in-vitro* techniques used for sperm washing. Pelleting sperm by cycles of centrifugation and resuspension induces ROS production [13].

## ROS AND SPERM FUNCTION

### Effects of physiological levels of ROS on sperm function

For proper functioning and fertility, mammalian sperm need to acquire certain properties including normal morphology, motility, and capability to undergo different events such as capacitation and acrosomal reaction. Research evidence has shown that physiological levels of ROS act as intracellular signaling molecules necessary for different physiological processes including maturation, hyperactivation, capacitation, acrosomal reaction, and oocyte-sperm fusion [4]. A recent study reported that ROS levels in the semen of fertile individuals using chemiluminescence assay in terms of the mean±standard deviation and median 25th to 75th percentiles were 0.01±0.02×10^6^ photons per minute (cpm)/20×10^6^ sperm and 0.009 (0.004–0.014)×10^6^ cpm/20×10^6^ sperm [14]. During sperm maturation, a low magnitude of OS is required for other physiological events including mitochondrial activity, enhanced zona binding of sperm ROS are required for the proper packing of chromatin material essential for stability [15].

Sperm maturation occurs inside the ductus epididymis involving different steps such as alterations in the plasma membrane, rearrangement of membrane proteins, enzymatic modulations, and nuclear remodeling [16]. All of the above-mentioned steps are regulated through the appropriate signaling pathways modulated by the ROS present in the seminal plasma [17,18]. ROS are also involved in the formation of disulfide bonds to ensure chromatic stability and prevent damage to the chromosomal DNA.

Sperm capacitation and hyperactivation are considered prerequisite events to ensure successful fertilization. Reports have indicated two main changes occurring at the cellular level responsible for sperm capacitation including the generation of physiological levels of ROS and phosphorylation of protein tyrosine. It was found that ROS induces phosphorylation as *in-vitro* inhibition of ROS by the introduction of 2-deoxyglucose resulted in a reduced concentration of tyrosine-phosphorylated proteins [19]. The process is triggered by an influx of calcium (Ca^2+^) and bicarbonate ions. According to Du Plessis and his coworkers [17], both Ca^2+^ ions and ROS are involved in the initiation of the capacitation to cause the activation of adenylate cyclase which results in the production of cyclic adenosine monophosphate (cAMP). cAMP further causes the activation of downstream protein kinase A (PKA). PKA stimulates a membrane-bounded NADPH oxidase resulting in enhanced ROS production and phosphorylates serine and tyrosine, which leads to the activation of protein tyrosine kinase. ROS not only promotes the protein tyrosine kinase but also inhibits phosphotyrosine activity that normally dephosphorylates tyrosine. Protein tyrosine kinase phosphorylates tyrosine present in the fibrous sheath surrounding the axoneme of sperm flagellum. The enhanced phosphorylation of tyrosine is observed in the capacitation. A-kinase anchoring proteins are phosphorylated proteins that play a role in binding PKA with the fibrous sheath of the sperm [20] suggesting their possible involvement in the hyperactivity of sperm.

Following capacitation, the acrosome reaction is considered the last step of sperm maturation to acquire fertility [17]. The acrosome reaction is initiated by zona pellucida (ZP), progesterone hormone, and ROS. Acrosome reaction results in the release of acrosomal enzymes mainly acrosine from the sperm head that helps sperm in penetrating the ZP of the oocyte. Release of Ca^2+^ ions from the acrosome results in the breakdown of phosphatidylinositol-4-5-biphosphate yielding diacylglycerol (DAG) and inositol triphosphate. Inositol triphosphate causes the activation of actin-serving proteins that facilitates the fusion of the acrosome and plasma membrane of sperm to trigger exocytosis. Whereas, DAG triggers protein kinase C (PKC) activation leading to a greater influx of Ca^2+^ ions and activation of phospholipase A2 to release large amounts of fatty acids from the plasma membrane required for the fusion of sperm with the oocyte [21,22].

#### Hydrogen peroxide

A proper level of H_2_O_2_ plays an important role in sperm function including sperm maturation, chromatin stability, capacitation, hyperactivation of sperm, and acrosome reaction, and increases the rate of sperm-oocyte fusion [23]. Furthermore, peroxides have been reported to be involved in the formation of a “mitochondrial capsule” that protects the mitochondria against proteolytic degradation [24]. Mitochondrial protection is essential for cellular metabolism, mediation of apoptosis, and ROS production. Recent reports have demonstrated that enhanced concentration of H_2_O_2_ is involved in sperm capacitation through the activation of adenylyl cyclase to produce cAMP which results in PKA-dependent phosphorylation of tyrosine residue [25]. According to Griveau and his co-workers, a 25 μM concentration of H_2_O_2_ enhanced sperm capacitation and hyperactivation following 3 h of incubation in B2 medium [26]. Contrary to the previous study, another study reported that a 50 μM concentration of H_2_O_2_ causes a twofold increase in cAMP production through adenylyl cyclase activation that leads to PKA-dependent protein tyrosine phosphorylation essential for capacitation [25].

#### Superoxide anion

O_2_^–•^ is involved in sperm maturation, capacitation, acrosomal reaction, and sperm-oocyte fusion by enhancing the membrane fluidity of sperm [18]. Moreover, O_2_^–•^ is the major species responsible for ROS-induced sperm hyperactivation possibly resulting from increased intracellular adenosine triphosphate (ATP) levels [27].

The role of O_2_^–•^ in sperm capacitation was evident by the fact that O_2_^–•^ produced during the incubation of sperm under capacitation conditions [28]. A study reported that during cryopreservation, O_2_^–•^ improved the percentage of capacitated bovine sperms demonstrated by the induction of acrosome reaction using lysophosphatidylcholine [29]. Similar findings were reported by Zhang and Zheng [30] that exogenous O_2_^–•^ significantly improved the percentage of human sperm that underwent capacitation (from 14.0±1.3 to 23.2±2.5) and acrosome reaction (from 4.5%±1.1% to 16%±2.0%, respectively). However, another study reported that no increase in the spontaneous acrosome reaction was observed following a direct addition of O_2_^–•^ to the medium [21]. The presence of O_2_^–•^ resulted in the production of unesterified fatty acids from the membranal phospholipids. Based on these findings it was suggested that O_2_^–•^ secreted by sperm could be responsible for the ionophore-induced acrosome reaction via the de-esterification of membranel phospholipids [31].

#### Nitric oxide

Nitric oxide (NO^•^) is a free radical with a relatively long half-life. NO^•^ production is catalyzed by nitric oxide synthase. NO^•^ has been identified in the endothelium of testicular blood vessels. Physiological levels of NO^•^ actively participate in signal transduction pathways responsible for sperm motility, capacitation, and acrosomal reaction and could stimulate the hyperactivation of mouse sperm [32].

NO^•^ reported controlling sperm motility, at a low concentration of NO^•^, an increase in sperm motility has been observed. Whereas, moderate to high concentrations inversely affect sperm motility [33]. Hellstrom and his coworkers reported that a low level of sodium nitroprusside, a NO^•^ producing compound improved sperm motility and viability due to reduced lipid peroxidation (LPO) [34]. Similar findings were observed in another study that low concentrations of sodium nitroprusside (10^–7^ and 10^–8^ M) significantly improved the percentage of capacitated sperm following 3 h of incubation [35]. Regulation of enzymatic activity might be the reason for the improved capacitation rate of sperm.

Furthermore, NO^•^-releasing compounds trigger the capacitation of human sperm but the effect on hyperactivation was not constant [36]. The effect of NO^•^ on sperm capacitation was might be due to the oxidation of cellular components including membrane lipids or thiol groups either due to its direct reaction with H_2_O_2_ that leads to the formation of singlet oxygen or due to the oxidation of NO^•^ to form nitrosonium cation that can react with H_2_O_2_ to yield peroxynitrite anion [37].

Research evidence has shown that NO^•^ improves the binding of sperm with ZP. A study demonstrated that sperm treated with low concentrations of sodium nitroprusside (10^–7^ and 10^–8^ M) resulted in a significantly improved percentage of sperm binding with ZP following 3 h of incubation [35]. The effect of NO^•^ on sperm-oocyte binding may be due to the interaction with H_2_O_2_ and O_2_^–•^.

### Effects of pathological levels of ROS on sperm function

Male infertility may be associated with excessive ROS production in semen. Excessive ROS production overcomes the antioxidant’s defense systems, disrupting the natural balance between ROS production and neutralization by antioxidants resulting in OS. In infertile individuals, ROS levels examined in the sperm samples in terms of the mean±standard deviation and median 25th to 75th percentiles were 0.35±0.67×10^6^ cpm and 0.06 (0.02–0.33)×10^6^ cpm/20×10^6^ sperm [14]. OS produces pathological defects in major biomolecules including lipids, nucleic acids, proteins, and sugars [16]. The magnitude of oxidative damage depends on different factors including the nature and amount of ROS, duration of ROS exposure, temperature, oxygen tension, and composition of the surrounding environment (ions, proteins, and antioxidants) [38].

#### Lipid peroxidation and motility reduction

The plasma membrane of mammalian sperm is rich in PUFAs; fatty acids with more than two carbon-carbon double bonds [38]. These unconjugated double bonds are present between the methylene groups of PUFAs. The double bond near the methylene group reduces the strength of the methylene carbon-hydrogen bond, increasing hydrogen’s susceptibility to oxidative damage [39]. OS leads to a cascade of chemical reactions known as LPO. LPO is regarded as an autocatalytic self-propagating reaction that results in abnormal fertilization; it results in the loss of 60% of the fatty acids present in the plasma membrane, inversely affecting membrane fluidity, enhancing the permeability of ions, and inhibiting the actions of enzymes and receptors, as well as compromising sperm membrane integrity, defective motility, and reduced sperm-oocyte interaction [39].

#### DNA damage and apoptosis

OS causes severe damage to the nuclear material of sperm resulting in enhanced DNA fragmentation, modifications of base–pairs, chromatin cross-linking, and chromosomal microdeletions [11]. DNA damage results in cellular apoptosis, reduced fertilization rate, a higher percentage of miscarriage, and offspring mortality [40]. ROS-induced oxidative damage is also responsible for mutations in mitochondrial DNA that inversely affect sperm motility by inhibiting energy production. Agarwal et al [11] reported the involvement of at least one mitochondrial gene (of the 13) that codes for the electron transport chain system for reducing ATP and inducing intracellular ROS production. In infertile individuals, mature sperm are associated with higher ROS levels resulting in a significantly higher percentage of sperm undergoing apoptosis compared to the mature sperm of healthy individuals [41]. Reports have indicated a higher level of cytochrome C in the seminal fluids of infertile individuals, reflecting severe mitochondrial damage [6,11].

## DEFENSE AGAINST ROS IN SEMEN

**Figure 1:**
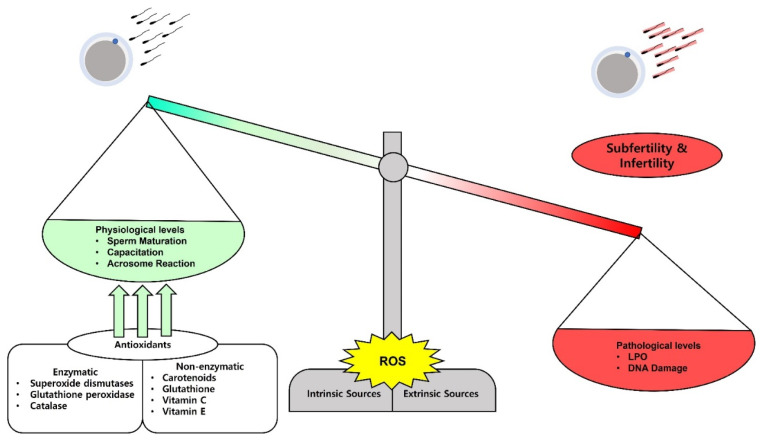
The balance and impacts of reactive oxygen species (ROS) on sperm fertilizing ability. ROS can be released physiologically and on a minimal level they play a role in sperm maturation, capacitation, and acrosome reaction. Paradoxically, high levels of ROS damage the sperm membrane lipids and the nuclear DNA and lead to subfertility and infertility. The balance can be adjusted by administration of several kinds of antioxidants to treat the subfertility and infertility cases.

Antioxidants are compounds or enzymes that can dispose of, scavenge/neutralize, and inhibit ROS production or their actions. Antioxidants help to maintain cell function and structure by protecting the plasma membrane against ROS. Furthermore, antioxidant protects acrosome integrity preventing premature acrosome reaction. Antioxidants work by breaking the oxidative chain reaction resulting in reduced OS. Antioxidants can protect sperm from ROS produced by abnormal sperm or leukocytes, prevent DNA fragmentation and premature sperm maturation, reduce cryodamage, and improve sperm quality. We provide a summarized figure to illustrate the balance in ROS production on the physiological and pathological levels (Figure 1).

During spermatogenesis, sperm lose most of the cytoplasmic contents rendering a very low intracellular antioxidant capacity. Therefore, sperm protection against ROS mainly depends on the antioxidant capacity of the seminal plasma. Seminal plasma serves as the main barrier against extracellular ROS, containing different enzymatic and non-enzymatic antioxidant molecules including CAT, carotenoids (vitamin A), coenzyme Q 10 (CoQ10), GSH, GPx, GSH reductase, pyruvate, SOD, taurine, hypotaurine, uric acid, vitamin C, and vitamin E [5]. The antioxidant system of the body is affected by the dietary intake of antioxidants, minerals, and vitamins [42]. The use of antioxidants to neutralize the overproduction of ROS either directly into the semen extenders or inclusion in the diet has been well-researched and reported in the literature. In general, dietary antioxidants demand long-term and more persistent treatment protocols to benefit male fertility. The effect of each antioxidant depends on the dosage used and species of animal involved. Similarly, to preserve the integrity of sperm during freeze-thaw procedures, multiple relationships and mechanisms have been established. However, insights into how antioxidants serve protection and energy to sperm are still paradoxical. In this section, we focus on the role of endogenous antioxidants in preserving sperm fertility (Tables 1, 2).

### Enzymatic antioxidants of seminal plasma

#### Superoxide dismutases

SODs are metalloenzymes present in all life forms. SODs are considered an integral part of the antioxidant defense system that plays an active role in protecting sperm against OS. There are two main SOD isoforms including SOD-1 (75% of antioxidants) and SOD-3 (25% of antioxidants) that are derived from the prostate [43]. SOD protect cells against excessive O_2_^–•^ levels by catalyzing the conversion of two O_2_^–•^ into molecular oxygen and H_2_O_2_ [44]. Based on the presence of transition metal ions at the active site, SODs are classified into four main types: copper/zinc SOD, iron SOD, manganese SOD, and nickel SOD [45]. Peeker et al [43] reported that copper/zinc SOD is predominantly found in both sperm and seminal plasma.

In the male reproductive tract, SODs are secreted into the seminal plasma by the accessory sex glands, epididymis, sperm, and testicles (Sertoli and Leydig cells) and help maintain sperm motility for a long period [46]. Several reports have indicated that a SOD-supplemented semen extender could improve the freeze-thaw quality of bull [47] and stallion sperm [48]. In another study, supplementation of the canine freezing extender with SOD, CAT, and GPx preserved the quality of sperm obtained from fertile and sub-fertile dogs for 10 days at 4°C [49].

#### Glutathione peroxidase

GPx is an important enzyme responsible for the detoxification of any lipid peroxide. GPx utilizes GSH as an electron donor to catalyze the reduction of H_2_O_2_ and O_2_^–•^ [50]. GPx is regarded as superior to CAT in maintaining low cellular H_2_O_2_ [44]. The active site of GPx contains selenium in the form of selenocysteine [10]. GPx is found in both sperm and seminal plasma. In sperm, GPx is primarily located in the mitochondrial matrix whereas seminal GPx is suspected to originate from the prostate [51]. Moreover, GPx is expressed and secreted from the epididymal head into the semen [46]. The primary function of GPx is to protect the sperm plasma membrane against LPO, and sperm DNA from oxidative damage and chromatin condensation [50].

#### Catalase

CAT is an enzyme found in the peroxisomes that decompose H_2_O_2_ into water and an oxygen molecule to prevent LPO of the plasma membrane. In semen, CAT was reported to be present in both sperm and seminal plasma. Seminal plasma is considered the main source of CAT; however, developing sperm also show a minimal level of CAT [52]. It was believed that CAT in the seminal plasma originated from the prostate gland [53]. The importance of CAT in seminal plasma is evident based on the observation that the semen of asthenozoospermic individuals may contain lower levels of CAT than that of normospermic individuals [46].

CAT supplementation reduced ROS levels and cryodamage in freeze-thaw sperm samples [54]. Moubasher et al [55] reported that supplementing fresh and processed semen with CAT results in improved freeze-thaw sperm motility, viability, and DNA integrity. Similarly, a CAT-supplemented semen extender prolonged sperm survival in camels [56]. Similar results were reported in another study when the freezing extender was supplemented with both CAT and SODs [57]. It was suspected that improvement in the freeze-thaw sperm quality was attributable to the combined and simultaneous action of both antioxidants against O_2_^–•^ and H_2_O_2_ [57].

### Non-enzymatic antioxidants of seminal plasma

#### Carotenoids (Vitamin A)

Carotenoids are fat-soluble organic compounds. Being precursors of vitamin A, carotenoids are mainly found in different vegetable dyes including orange, pink, red, and yellow. Carotenoids such as beta-carotenoids and lycopene are important components of the antioxidant defense system. Carotenoids help maintain the integrity of plasma membranes, regulate the proliferation of epithelial cells, and actively participate in spermatogenesis [58]. A carotenoid-deficient diet can lead to reduced sperm motility [10].

#### Reduced glutathione

GSH is a natural antioxidant found in reproductive tract fluids and epididymal sperm semen of most of animal species and acts as a substrate in the peroxidase/reductase pathway to maintain the equilibrium and protect sperm from oxidative damage [59].

Reports have indicated that supplementation with GSH and its precursors (cysteine and glutamine) resulted in improved semen quality. In 1996, Irvine reported that the use of GSH for the treatment of infertile individuals with a varicocele or inflamed urogenital system resulted in significantly improved sperm quality [60]. In another study, a GSH-supplemented freezing extender improved the motility of donkey sperm by reducing the intracellular ROS levels [59]. However, GSH supplementation did not significantly affect other parameters of donkey sperm including plasma and acrosomal membrane integrity, mitochondrial membrane potential (MMP), and intracellular O_2_^–•^ levels. Olfati Karaji and his co-workers reported improved freeze-thaw sperm quality by using a combination of GSH and SOD in the freezing extender of bull [61]. It was suspected that improved sperm quality was associated with reduced LPO and enhanced antioxidant levels.

#### Cysteine

Cysteine is a GSH precursor that can restore GSH depletion because of OS and inflammation [62]. In a clinical trial, oral intake of cysteine (600 mg/d) for 3 months improved the sperm quality and antioxidant status of infertile men [63]. Another report indicated that incubation of human sperm with cysteine for 2 h at room temperature significantly improved sperm motility [64]. Moreover, a cysteine-supplemented freezing extender protected sperm during the freeze-thaw procedure and resulted in improved sperm quality in bull [65], chicken [66], and ram [67] sperm.

#### Vitamins C and E

Vitamin C is a naturally occurring water-soluble substance having outstanding antioxidant properties. Vitamin C protects sperm against oxidative damage by neutralizing O_2_^–•^, H_2_O_2_, and OH^•^ [68]. Moreover, Vitamin C could effectively protect sperm DNA from ROS because of its high antioxidant competency [68]. The concentration of vitamin C is 10 times higher in seminal plasma than in the blood (364 vs 40 μmol/L) [10].

Several *in-vivo* studies have been performed to investigate the therapeutic potential of vitamin C for the restoration of fertility. Dawson et al [69] observed a positive correlation between vitamin C levels and sperm quality-related parameters including sperm concentration, motility, and viability. In that study, oral administration of vitamin C in smokers significantly improved the vitamin C levels in the seminal plasma and serum, resulting in improved semen quality. Similar findings were reported by Akmal et al [70] in infertile men with idiopathic oligozoospermia treated with vitamin C (2 gm/d). In another study, vitamin C supplementation (250 mg twice a day) resulted in improved sperm motility and morphology in patients following the surgical removal of varicocele [71]. However, vitamin C failed to improve the sperm count in such individuals. Vitamin C supplementation could effectively restore the fertility of rats with cyclophosphamide-induced testicular OS and androgenic disorders [72].

Furthermore, several *in-vitro* studies have reported improved sperm quality following the use of mediums supplemented with vitamin C. Inclusion of vitamin C (800 μmol/L) in the Ringer-Tyrode medium protected sperm from ROS-induced damage and improved sperm motility and viability [73]. However, higher concentrations of vitamin C (e.g. 1,000 μM) instead of protecting sperm against H_2_O_2_ increased the magnitude of oxidative damage. In another study, the supplementation of Percoll medium with vitamin C (600 μM) protected sperm DNA from damage [74]. Similar findings were obtained by another study where vitamin C-supplemented TEST yolk buffer failed to preserve sperm motility [31].

Vitamin E is a naturally occurring fat-soluble compound. Vitamin E is mainly present in the plasma membrane and possesses powerful chain-breaking antioxidant properties with dose-dependent effects. Vitamin E neutralizes free OH^•^ and O_2_^–•^ anions in the plasma membrane and reduces ROS-induced LPO. Therefore, vitamin E mainly protects the components of the sperm plasma membrane against LPO and improves the function of other antioxidants.

Different *in-vivo* studies have shown that vitamin E could be effectively used for the treatment of infertile individuals with oligoasthenozoospermia induced by OS [75,76]. Suleiman et al. observed that oral intake of vitamin E significantly increased the motile sperm count by decreasing malonic dialdehyde (end product of LPO) production from sperm [77]. In another study, Eid et al [78]. observed that vitamin E supplementation resulted in improved sperm concentration, motility, viability, and enhanced oxidative function in the seminal plasma of chickens [78].

Similarly, *in-vitro* studies have reported that vitamin E preserves sperm motility and also enhances their ability to penetrate hamster eggs [79]. During freeze-thaw procedures, the use of vitamin E-supplemented semen extenders at an inclusion rate of 10 mmol/L preserved sperm motility more efficiently compared to an untreated control group [31]. In 2003, Park and his coworkers reported that vitamin E supplementation resulted in reduced sperm damage and improved sperm motility during freeze-thaw procedures [80]. In another study, vitamin E-supplemented Percoll medium protected sperm DNA against oxidative damage [74].

Recent studies have shown that combined use of vitamins C and E together or alongwith other antioxidants can effectively improve semen quality. The oral intake of vitamins C and E can greatly reduce ROS-induced DNA damage in the sperm of normozoospermic and asthenozoospermic men [81]. Similarly, Greco et al. also observed reduced sperm DNA damage in infertile individuals following combined supplementation with vitamin C and vitamin E for 2 months [82]. It is believed that the use of hydrophilic vitamin C along with the lipophilic vitamin E results in a synergistic effect that reduces the magnitude of sperm damage induced by OS [83]. Similar findings were observed when vitamin C was used together with vitamin E and GSH [84]. Moreover, vitamin E combined with other antioxidants including β-carotene [85], vitamin C, GSH [86], and selenium [76,87] led to an improved semen profile in infertile individuals.

#### Taurine and hypotaurine

Taurine is a sulfur-containing amino acid that protects sperm against ROS when exposed to aerobic conditions or freeze-thaw procedures [88]. The antioxidant nature of taurine is related to its ability to elevate the CAT level in close association with the SOD concentration in bull, ram, and rabbit sperm [88].

An *in-vivo* study indicated that taurine use can significantly reverse the toxic effects of endosulfan in rats. Taurine treatment improved testicular weight, sperm count, motility, viability, and daily sperm production in endosulfan-treated rats [89].

An *in-vitro* study indicated that taurine and hypotaurine stimulate sperm capacitation and acrosomal reaction [90]. Furthermore, hypotaurine and taurine can inhibit spontaneous LPO in epididymal sperm [91]. Boatman et al [92] reported that hypotaurine restored the motility of hamster sperm affected by the washing procedure. Several studies have utilized taurine-supplemented semen extenders during different storage procedures. Storage of ram semen at room temperature using a taurine-supplemented extender significantly improved motility, membrane integrity, antioxidant status, and total antioxidant capacity [93]. Moreover, a taurine-supplemented semen extender showed the same protective effect during the chilling of tom [94], stallion [95], and donkey [96] sperm. Taurine supplementation resulted in improved freeze-thaw motility, viability, and plasma membrane integrity of buffalo [88], bull [97], and ram [98] sperm.

#### Coenzyme Q10

CoQ10 is a vitamin-like substance synthesized from tyrosine and serves as an important component of the inner mitochondrial membrane, an energy-promoting agent by supporting the mitochondrial electron transport chain, present in the mid-piece of the sperm tail. CoQ10 neutralizes O_2_^–•^ and peroxides to protect lipids from oxidative damage. Gvozdjáková et al [99] reported that CoQ10 works by regenerating other antioxidants including vitamin E and vitamin C.

Several clinical trials have shown that CoQ10 supplementation resulted in improved semen quality in infertile individuals [100]. A meta-analysis of clinical trials investigating the effects of CoQ10 supplementation showed a significant improvement in sperm motility (total and progressive), sperm concentration, and seminal concentration of CoQ10 [101].

Some clinical trials have reported the beneficial effects of combined use of CoQ10 with other antioxidants. In male rats, oral intake of CoQ10 and L-carnitine attenuated the effects of high and oxidized low density lipoprotein (LDL) resulting in a significantly improved hormonal profile and sperm quality [102]. These improved outcomes may be attributable to efficient energy production from sperm mitochondria, which requires sufficient concentrations of CoQ10 and carnitine [99]. Gvozdjáková et al [99] reported that daily intake of CoQ10 (30 mg), L-carnitine (440 mg), vitamin C (12 mg), and vitamin E (75 IU) improved sperm concentration and pregnancy rates in infertile individuals.

*In-vitro* studies have shown the protective role of CoQ10 during freeze-thaw sperm procedures [103–105]. A CoQ10-supplemented freezing extender reduced the magnitude of cryodamage and resulted in the improved freeze-thaw quality of buck [106], fish [103], and ram [107] sperm. Similar results were observed when boar semen was stored at 17°C [108] and rooster semen was stored at 5°C [104] after being diluted with a CoQ10-supplemented semen extender. In contrast, a recent study showed that the addition of CoQ10 in the freezing extender of stallions did not affect the freeze-thaw sperm quality, but oral supplementation of stallions resulted in improved motility and membranal integrity of sperm after 24 h of cooling [109]. Similar findings were observed in another study where stallions were orally fed a diet supplemented with CoQ10 (1 gm/d) and improved semen quality was observed in the semen of five out of seven stallions following the cooling and freezing of semen [110].

## EXOGENOUS ANTIOXIDANTS AND SPERM FERTILITY

Under normal conditions, endogenous antioxidant systems are primarily involved in the regulation of redox control. However, certain pathological conditions are associated with excessive ROS production overcoming redox control. In such circumstances, antioxidants from exogenous sources can play an important role in ameliorating the detrimental effects of OS. In this section, we focus on the role of exogenous antioxidants in preserving fertility (Table 3).

### Astaxanthin

Astaxanthin (AXN) is a red keto-carotenoid pigment that has shown antioxidant activity against different oxidants and can inhibit LPO by penetrating biological membranes as well as suppresses ROS-induced damage to DNA, lipids, and proteins [111].

Several studies have reported that AXN has positive effects on fertility. An AXN-supplemented diet improved the osmolality, motility, concentration, and fertilization rate of sperm in goldfish [112]. In a clinical trial, Comhaire et al [113] reported that oral intake of AXN had a positive impact on semen quality and fertility of infertile individuals. In another study, oral intake of AXN combined with vitamins C and E ameliorated infertility in male rats [114]. AXN supplementation was reported to ameliorate the detrimental effects of diabetes on sperm parameters in rats [115].

Recent reports have confirmed the protective role of AXN during sperm preservation. AXN supplementation showed improved and protected sperm motility, viability, membrane integrity, and DNA during liquid preservation of boar [115] and ram [116] semen. Similar findings were observed during freeze-thaw procedures using an AXN-supplemented freezing extender in boar [117], dog [118], and ram [119] sperm.

### Kinetin

Kinetin, a member of the cytokinin family has positive effects on cellular growth and division by reducing cycle length. Previous reports have indicated that kinetin can regulate the antioxidant activities of enzymes including CAT and SOD [120] resulting in reduced oxidative damage and is reported to reduce oxidative damage during *in-vitro* cell culture [121]. Recently, kinetin use was shown to be effective in alleviating cisplatin-induced testicular toxicity and organ damage by reducing OS, inflammation, and apoptosis [122]. During freeze-thaw procedures, the use of a kinetin-supplemented freezing extender resulted in improved sperm motility, viability, and structural integrity of dog [123] and ram [124] sperm.

### Myo-inositol

Myo-inositol (MYO) is the most important naturally existing inositol and belongs to vitamin B complex group 1. MYO regulates the intracellular level of calcium ions and it has been suggested that MYO has a role in spermatogenesis and sperm function. Sertoli cells secrete MYO in response to the follicle-stimulating hormone that regulates different physiological events associated with sperm including maturation, motility, capacitation, and acrosomal reaction [125].

MYO has the potential to restore the fertility of male gametes and improve the fertilization rate [126]. In a clinical trial, oral intake of MYO resulted in improved sperm quality and balanced hormonal profiles in patients with idiopathic infertility [127]. Condorelli et al. suggested the use of MYO in infertile individuals based on the findings of their study that incubation of sperm in a medium supplemented with MYO (2 mg/mL) reduces the percentage of sperm with low MMP [128]. In another study, Condorelli et al. reported that MYO enhanced the motility of sperm retrieved following the swim-up procedure in both fertile and infertile individuals [129]. Furthermore, an MYO-supplemented freezing extender showed reduced OS and improved freeze-thaw sperm quality in different species including dogs [130], fish [131], bucks [132], and humans [133]. Similar results were observed when MYO supplementation was used during thawing procedures [134].

### Quercetin

Quercetin (QR) is a flavonoid derived from plants and vegetables with strong antioxidant properties owing to the presence of three OH^•^ groups. QR has been used to treat male infertility issues by scavenging ROS, as QR-supplemented sperm showed low levels of H_2_O_2_ [135]. Johinke et al [135] reported that sperm medium supplemented with QR protected against OS in 15°C-stored rabbit sperm over 96 h period. Moreover, recent studies have confirmed the protective role of QR against oxidative damage during the freeze-thaw procedure, as QR-supplemented freezing extender induced significant quality improvement in buck [136], bull [137], dog [138], human [139], and stallion [140] sperm.

### Selenium

Selenium is an essential component of a specific group of proteins known as selenoproteins. It is believed that the antioxidant nature of selenium is related to its ability to enhance GSH function. Selenium plays a major role in spermatogenesis and sperm maturation [141] and can protect sperm from ROS-induced DNA damage. The deficiency of selenium leads to certain defects such as mid-piece abnormalities and abnormal sperm motility [142]. Incubation of sperm from asthenoteratozoospermic individuals in a selenium-supplemented medium enhanced the percentage of motile sperm, sperm viability, and MMP [143]. Furthermore, selenium supplementation decreased LPO and DNA fragmentation.

### Zinc

Zinc (Zn^2+^) is an essential trace element that stimulates total antioxidant status, it helps reduce the production of H_2_O_2_ and OH^•^ radicals through the neutralization of redox-active transition metals, such as iron and copper [144]. A recent study showed that Zn^2+^ can decrease DNA damage caused by the addition of H_2_O_2_ [145]. Fertile men have a significantly higher level of Zn^2+^ in the seminal plasma than subfertile men [146].

Zn^2+^ has a protective effect on sperm structure, as its deficiency leads to different tail defects including hypertrophy and hyperplasia of the fibrous sheath, axonemal disruption, defects of the inner microtubular dynein arms, and abnormal or absent mid-piece [147]. An *in-vitro* study reported that 10 μg/mL is the optimum concentration of Zn^2+^ that positively affects total and progressive sperm motility along with a reduction in DNA fragmentation and LPO [148]. Berkovitz et al [149] reported that Zn^2+^ supplementation before sperm freezing had beneficial effects on sperm motility and viability. They also observed improved sperm motility in freeze-thaw and in semen samples refrozen after thawing [149].

### Sericin

Sericin is a glue-like structure with strong antioxidant properties. Silkworm covers the silk filament with sericin to connect filaments and provide protection against a harmful environment. Recently, sericin has been used for liquid storage and freezing of sperm in different species including buck [150], bull [151], rabbit [152], and stallion [153]. Sericin supplementation resulted in an improved freeze-thaw sperm quality through an improved antioxidant status and reduced ROS level.

## CONCLUSION

In the male reproductive system, ROS production is associated with different physiological and pathological conditions. ROS overproduction negatively influences fertility by disturbing the natural balance between ROS production and neutralization. OS-induced infertility appears to be a major challenge. Over the years, different antioxidant therapies have been utilized to address the infertility issues associated with OS either in the form of oral consumption or as *in-vitro* supplementation of different mediums. However, the outcomes of such studies appear to be controversial making it difficult to draw a conclusion. The reasons may include the low sample size used, differences in the concentrations used, and issues with the experimental designs. Moreover, the failure of antioxidants therapy can be attributed to the lack of real-time assessment methods and the inability to accurately quantify seminal OS.

To achieve better results, studies should be performed using larger sample sizes, classical pharmacological concentrations, and better-designed experiments. Furthermore, fertility can be recuperated using a combination of remedies (antioxidants, vitamins, trace minerals) along with knowledge of the underlying cause and severity of infertility. A new combination of antioxidants especially with polyphenols has shown a massive potential to treat infertility. Moreover, ROS levels in infertile individuals should always be correlated with the microenvironment of semen and reproduction outcomes (conception rate, quality of sperm functions, and embryo). This database will help in the development of reliable assays for the assessment of OS in reproductive cells and fluids.

## Figures and Tables

**Table 1:** Effects of endogenous-enzymatic antioxidants on male fertility

Antioxidant	Study type	Specie	Dose used	Outcome	Reference
SOD	*In-vitro*	Stallion	25, 50 U/mL	Improved progressive motility and viability of sperm	[48]
CAT	*In-vitro*	Human	200 U/mL	Higher progressive motile sperm Reduced DNA-damaged sperm	[55]
		Camel	500 IU/mL	Higher total and progressive motile sperm	[56]
GPx, SOD	*In-vitro*	Bull	0.0, 0.5, 1.0 mM 0, 250, 500 U/mL	Non-return rates are 74% and 73.9% Improved sperm motility (6% to 11%)	[154]
SOD, CAT, GPx	*In-vitro*	Dog	15 IU/mL	Higher progressive motile sperm Significantly higher DNA integrity	[49]
Ascorbate & CAT	*In-vitro*	Human	300, 600 μM 200, 400 U/mL	Reduced significantly ROS levels in post-thaw sperm	[54]
SOD, CAT	*In-vitro*	Human	200 U/mL	Significantly improved sperm quality Reduced LPO	[57]

SOD, superoxide dismutase; CAT, catalase; GPx, glutathione peroxidase; ROS, reactive oxygen species; LPO, lipid peroxidation.

**Table 2:** Effect of endogenous non-enzymatic antioxidants on male fertility

Antioxidant	Study type	Specie	Dose used	Outcome	Reference
GSH	*In-vitro*	Donkey	0, 2, 4, 6, 8, 10 mM	Significantly higher motility & kinematic parameters Higher sperm viability	[59]
	*In-vivo*	Human	600 mg	Significantly improved fertility	[60]
Reduced GSH & SOD	*In-vitro*	Bull	GSH: 5, 7.5 mM SOD: 50, 100 U/mL	Improved total and progressive motility parameters	[61]
Cysteine	*In-vivo*	Human	600 mg	Significantly improved sperm count and motility Significantly decreased abnormal morphology DNA fragmentation and protamine deficiency	[63]
	*In-vitro*	Human	1.0 mg/mL	Significantly improved sperm function and motility Significantly decreased ROS production	[64]
		Buffalo	0.0, 0.5, 1.0, 2.0, 3.0 mM	Improved antioxidant status, freeze-thaw quality, and in-vivo fertility	[65]
Cysteine & SOD	*In-vitro*	Chicken	5 mM 200 U/mL	Prevented the reduction in motility, viability, and mitochondrial membrane potential Protected sperm against apoptotic changes	[66]
Cysteine & Erythioneine	*In-vitro*	Ram	0, 1, 2, 4 mM	Improved freeze-thaw sperm motility and mitochondrial activity	[67]
Vitamin C	*In-vivo*	Human	5, 10, 20, 60, 250 mg/d	Reduced oxidative DNA damage of sperm	[68]
			50, 100, 200, 400, 800, 1,000, 2,000, 4,000 μg	Improved sperm motility and viability through reduced ROS production	[73]
			0, 200, 1,000 mg/d	Improved sperm quality	[69]
			1,000 mg/d	Significant rise in sperm count, sperm motility, and sperm with normal morphology	[70]
			500 mg/d	Improved sperm motility and morphology	[71]
		Rat	25 mg/kg/d	Protective role on cyclophosphamide-induced testicular dysfunction Alleviates the cyclophosphamide-induced OS	[72]
			0.88 mg/kg	Higher sperm motility, viability, and count	[155]
Vitamin E	*In-vivo*	Chicken	0, 20, 40, 80, 160 mg/kg	Higher sperm viability and motility (40–160 mg/kg) Higher sperm concentration (80 mg/kg)	[44]
			200 mg/kg diet	Increased both sperm count and motility Reduced percentage of dead sperm	[78]
		Human	600 mg/d	Significant improvement in in-vitro sperm function	[75]
			100 mg/d	Significantly higher sperm motility Significantly decreased MDA levels	[77]
Vitamins C & E	*In-vivo*	Human	1,000 mg/d 800 mg/d	Increased ejaculate volume, sperm count, and motility	[81]
			1,000 mg/d	Markedly reduced DNA-fragmented sperm	[82]
	*In-vitro*	Human	10 mM	Minimum improvement in sperm motility	[31]
Vitamins C & E, CAT, Hypotaurine, Cysteine, GSH	*In-vitro*	Human	0.2 and 1 mM 2,600 U/mL 1 & 10 mM 1 & 10 mM 1 & 10 mM	Protects against OS	[83]
Vitamins C & E, GSH	*In-vitro*	Human	200 mg, 200 mg, 400 mg	Reduced OS	[84]
Vitamins C & E, Urate, Cysteine	*In-vitro*	Human	300, 600 μM 30, 60 μM 200, 400 μM 5, 10 μM	Improved sperm DNA integrity	[74]
Mesterolone & Vitamin C	*In-vivo*	Human	50 mg 200 mg	No improvement in male fertility	[86]
Cysteine, Vitamin A, & Vitamin E	*In-vivo*	Human	600 mg/d 30 and 180 mg/d	Neutralized the ROS and improve sperm count No effect on sperm motility	[85]
Selenium	*In-vivo*	Human	100 μg/d	Significant rise in plasma selenium concentration and sperm motility	[156]
			400 U/d 200 μg/d	Significantly higher sperm motility, viability, and fertility	[87]
			400 mg/d 225 μg/d	Significantly improved sperm motility Significantly decreased MDA levels	[76]
Rebamipide	*In-vitro*	Human	10, 30, 100, 300 μM	Decreased ROS level and LPO No effect on sperm viability	[80]
Taurine	*In-vitro*	Sheep	0, 10, 20, 40, 80, 100 mM	Reduced LPO and improved semen parameters	[93]
		Cat	50 mM	Higher sperm motility, Reduced abnormal sperms with sperm defects	[94]
		Stallion	70, 100 mM	Improved sperm survival	[95]
		Donkey	0, 20, 40, 60 mM	Significantly improved sperm motility	[96]
		Hamster	0, 2×10^−3^, 2×10^−4^, 2×10^−5^ M	Improved motility and promote capacitation	[90]
Hypotaurine	*In-vitro*	Hamster	10 mM	Restore sperm motility and viability	[92]
Taurine & Hypotaurine	*In-vitro*	Rabbit	0.5 mM	Reduced LPO and restored motility	[91]
Taurine & Trehalose	*In-vitro*	Cattle Bull	50 mM 100 mM	Significantly improved freeze-thaw motility, viability, and plasma membrane integrity	[97]
		Buffalo Bull	50 mM 100 mM	Improved motility, viability, and membrane integrity Low numbers of capacitated sperm	[88]
Taurine, QR, & GSH	*In-vitro*	Ram	40 mM, 5 μg/mL, 5 mM	Reduced LPO Improved freeze-thaw semen quality	[98]
CoQ10	*In-vivo*	Human	200 mg/d	Improved sperm concentration, progressive motility, total motility, and semen antioxidant status Reduced ROS level and SDF percentage	[100]
		Stallion	1 gm/d	Improved semen quality	[110]
	*In-vivo* & *In-vitro*	Human	5, 50 μM 60 mg	Improved sperm motility and fertilization rate	[157]
		Buffalo bull	30 Mm	Improved sperm parameters and fertility	[105]
		Rooster	0, 1, 2, 5, 10 μM	Significantly higher total sperm, progressive motilities, membrane functionality, viability, and mitochondria active potential Reduced LPO	[104]
		Buck	0, 0.5, 1, 1.5 μM	Improved sperm motility, viability, and plasma membrane functionality	[106]
		Giant grouper	0, 25, 50, 100 μM	Improved total sperm motility, fertilization rate, and reduced DNA fragmentation	[103]
		Boar	-	Increase the sperm characteristics and prolong the survival of liquid storage of sperm	[108]
		Stallion	40 & 80 μg/mL	No notable effect of semen quality	[109]
CoQ10 & L-carnitine	*In-vivo*	Rat	10 mg/kg/d 350 mg/kg/d	Significantly improved sperm quality and hormonal profile by weakening the high and oxidized LDL	[102]
CoQ10 & Ellagic acid	*In-vitro*	Ram	0.5 μM 0.25 mM	Higher total sperm, progressive motility, and viability No effect of sperm antioxidant level	[107]

GSH, glutathione; SOD, superoxide dismutase; BSA, bovine serum albumin; LPO, lipid peroxidation; ROS, reactive oxygen species; OS, oxidative stress; MDA, malondialdehyde; QR, quercetin; Q10, coenzyme; SDF, sperm DNA fragmentation; LDL, low density lipoprotein.

**Table 3:** Use of exogenous antioxidants to improve the fertility of males

Antioxidant	Route	Specie	Dose used	Outcome	Reference
AXN	*In-vivo*	Human	0, 16 mg/kg	Positive effect on sperm parameters and fertility	[113]
			0.5, 1, 2 μM	Inhibited LPO	[158]
		Ram	0, 0.5, 1, 2, 4 μM	Improved sperm vitality and membrane integrity Significantly reduced ROS production	[116]
		Rat	720 mg/kg	Improved sperm viability, normal morphology, and DNA integrity	[115]
	*In-vitro*	Boar	0, 0.5, 1, 2, 5 μM	Improved freeze-thaw semen quality, inhibited LPO	[117]
		Dog	0, 0.5, 1, 2 μM	Improved freeze-thaw sperm quality	[118]
		Ram	0, 0.5, 1, 2, 4 μM	Decreased acrosome abnormalities Improve semen quality and fertility rate	[119]
β-carotene & AXN	*In-vivo*	Goldfish	50, 100, 150 mg/kg	Improved osmolality, motility, fertilization rate, and sperm concentration	[112]
AXN & Vitamins E and C	*In-vivo*	Rat	100 mg/kg 100, and 200 mg/kg	Improved fertility	[114]
Kinetin	*In-vivo*	Wheat Seedling	1 μM	Improved growth, antioxidant, and chlorophyll content	[120]
		Rat	0.25, 0.5, 1 mg/kg	Reduced organ damage and OS Inhibited the apoptosis	[122]
	*In-vitro*	Dog	0, 25, 50, 100, 200 μM	Reduced OS and improved the freeze-thaw semen quality	[123]
		Ram	0, 25, 50, 100, 200 μM	Enhanced sperm kinematics, viability, plasma membrane functionality, and reduced LPO	[124]
MYO	*In-vivo* & *In-vitro*	Human	15 μL/mL	Improved sperm quality and motility	[126]
			2 g	Increased acrosome-reacted sperm, sperm concentration, and progressive motility Optimized serum LH, FSH, and inhibin	[127]
				No effect on mitochondrial function of sperm Increase sperm number with high MMP	[128]
				Increase sperm motility and sperm number retrieved after swim-up	[129]
				Improved total motility, progressive motility, and reduced DNA fragmentation Ineffective in inhibiting ROS level	[133]
			20 mg/mL	Improvement of sperm vitality and motility Reduction of ROS-induced sperm defects	[134]
		Dog	1, 2 mg/mL	Protected against OS and improved freeze-thaw sperm quality	[130]
		Catfish	5, 10, 20, 40 mg	Increased sperm motility, viability, and DNA integrity	[131]
MYO & Melatonin	*In-vitro*	Goat	-	Reduced ROS production, DNA damage, and LPO	[132]
	*In-vitro*	Rabbit	0, 25, 50, 100, 200 μM	Reduced H_2_0_2_ and LPO	[135]
			50 μM	Higher sperm motility, viability, and DNA integrity	[139]
		Goat	10, 20 μM	Improves freeze-thaw sperm motion characteristics and inhibited LPO	[136]
		Bull	25, 50, 100, 200 μg/mL	No beneficial effect of motility, plasma membrane integrity, and sperm defects	[137]
		Dog	0, 25, 50, 100 μM	Reduced oxidative damage and improved the semen quality	[138]
			0.15 mM	Improved sperm motility and zona binding ability, reduced DNA fragmentation	[140]
Selenium	*In vitro*	Human	2 μg/mL	Enhanced motility, viability, and MMP	[143]
Zn2+	*In-vitro*	Human	12.5 nM	Improved sperm parameters, and reduced DNA damage	[145]
			50 μM	Enhanced sperm total and progressive motility	[149]
Zn2+, D-aspartate, CoQ10	*In-vitro*	Human	1, 10, 100 μg/mL 5, 50, 500, 5,000 μg/mL 4, 40, 400 μg/mL	Improved progressive sperm motility, reduced DNA fragmentation	[148]
Sericin	*In-vitro*	Buck	0%, 0.25%, 0.5%	Ameliorated the freeze-thaw semen quality by improving the antioxidative status and minimizing the leakage of intracellular enzymes (0.25% Sericin)	[150]
		Bull	0%, 0.25%, 0.5%, 1.5%, 2%	Improved freeze-thaw semen quality by protecting against OS	[151]
		Rabbit	0%, 0.1%, 0.5%	Enhanced osmotic tolerance and freeze-thaw sperm quality, Reduces the ability of rabbit sperm cells to undergo in-vitro-induced acrosome reaction,	[152]
		Stallion	0.25%	Improved sperm DNA integrity and its resistance to ROS and LPO	[153]

AXN, astaxanthin; LPO, lipid peroxidation; OS, oxidative stress; MYO, myo-inositol; MMP, mitochondrial membrane potential; QR, quercetin; H_2_O_2_, hydrogen peroxide; Zn2+, Zinc; CoQ10, coenzyme Q10.
